# Quantifying Key Points of Hydraulic Vulnerability Curves From Drought-Rewatering Experiment Using Differential Method

**DOI:** 10.3389/fpls.2021.627403

**Published:** 2021-02-02

**Authors:** Xiao Liu, Ning Wang, Rong Cui, Huijia Song, Feng Wang, Xiaohan Sun, Ning Du, Hui Wang, Renqing Wang

**Affiliations:** ^1^Institute of Ecology and Biodiversity, School of Life Sciences, Shandong University, Qingdao, China; ^2^Shandong Provincial Engineering and Technology Research Center for Vegetation Ecology, Shandong University, Qingdao, China; ^3^Qingdao Forest Ecology Research Station of National Forestry and Grassland Administration, Shandong University, Qingdao, China

**Keywords:** calculated result, differential method, experimental result, hydraulic, loss of conductivity, water potential

## Abstract

Precise and accurate estimation of key hydraulic points of plants is conducive to mastering the hydraulic status of plants under drought stress. This is crucial to grasping the hydraulic status before the dieback period to predict and prevent forest mortality. We tested three key points and compared the experimental results to the calculated results by applying two methods. Saplings (*n* = 180) of *Robinia pseudoacacia* L. were separated into nine treatments according to the duration of the drought and rewatering. We established the hydraulic vulnerability curve and measured the stem water potential and loss of conductivity to determine the key points. We then compared the differences between the calculated [differential method (DM) and traditional method (TM)] and experimental results to identify the validity of the calculation method. From the drought-rewatering experiment, the calculated results from the DM can be an accurate estimation of the experimental results, whereas the TM overestimated them. Our results defined the hydraulic status of each period of plants. By combining the experimental and calculated results, we divided the hydraulic vulnerability curve into four parts. This will generate more comprehensive and accurate methods for future research.

## Introduction

Patterns of precipitation have substantially changed owing to global climate change, and in several parts of the world, the total precipitation has gradually decreased (Easterling et al., 2000; Högy et al., 2013; Gimbel et al., 2015; Ge et al., 2017; Oliveira et al., 2019). In this regard, the increase in drought severity and frequency has become a major driver of global forest mortality (Brodribb and Cochard, 2009; Anderegg et al., 2012; Liu et al., 2018; Oliveira et al., 2019).

Drought induced hydraulic failure, carbon starvation during prolonged stomatal closure, and lethal biotic attacks due to climate-mediated insect outbreaks and pathogens have been proposed as explanations of the tree dieback and mortality in water-limited environments (Adams et al., 2009; Sevanto et al., 2014; Liu et al., 2018). Hydraulic failure caused by embolism has been invoked as the most direct and critical mechanism that causes forest mortality (Martinez-Vilalta and Pinol, 2002; Nardini et al., 2013; O'Grady et al., 2013; Liu et al., 2018), which initially resulted in tree dieback and led to extensive tree death. Because tree dieback is the prelude to forest mortality, it is crucial to grasp the hydraulic status before the dieback period to predict and prevent forest mortality.

Tyree and Sperry (1988) proposed the concept of the hydraulic vulnerability curve (HVC), which can be used to quantitatively characterize hydraulic failure. The HVC describes the relationship between the loss of conductivity (*LC*) and the plant water potential. As a result, three key points derived from the HVC have been set up and are widely used in plant drought tolerance researches. The first is the air-entry point (Ψ_e_), it is an estimate of the xylem tension at which pit membranes are overcome within the conducting xylem and when cavitation starts, after which the *LC* begins to increase linearly (Sparks and Black, 1999; Domec and Gartner, 2001; Meinzer et al., 2009; Delzon and Cochard, 2014; Anderegg and Meinzer, 2015; Martin-StPaul et al., 2017; Torres-Ruiz et al., 2017). The second point is the fastest drop in the hydraulic conductivity (Ψ_m_), it is described as the steepest part of the vulnerability curve, and usually represents the embolism resistance (Meinzer et al., 2009; Corcuera et al., 2011; Zhang et al., 2016; Dulamsuren et al., 2018; Santiago et al., 2018; Dietrich et al., 2019; Kannenberg et al., 2019). The third point is the upper inflection point (Ψ_l_), it likely represents a lethal point and appears to be the value that reflects the inherent risk to critical hydraulic failure for most angiosperm (Choat et al., 2012; Scholz et al., 2014; Benito Garzón et al., 2018). Sperry et al. (1988) used the pressure with a 50% hydraulic conductivity loss (Ψ_50_) as an estimate of Ψ_m_. However, Pammenter and van der Willigen (1998) proved that Ψ_50_ was only an approximate value of Ψ_m_. Domec and Gartner (2001) estimated Ψ_e_ and Ψ_l_ with a pressure that causes 12% (Ψ_12_) and 88% (Ψ_88_) *LC*, respectively. However, it cannot be neglected that previous researches inferred the three key points from the vulnerability curves analysis, rather than through direct measurement. By combining the vulnerability curves and half-lethal effect, Hammond et al. (2019) studied the Ψ_l_ of *Pinus taeda* L., and they reported that Ψ_l_ of *P. taeda* has a pressure that can cause a 0.80 *LC*. This is different from the gymnosperms calculating point Ψ_50_ (Choat et al., 2012) and the global synthesis reported by Adams et al. (2017), in which the trees died when the hydraulic failure exhibited more than a 0.60 *LC* in all cases. Hammond et al. (2019) reported that different trees have variable points of no return. They strongly recommended that continued experimentation is necessary to assess the different tree species, populations, and individuals in different ontogeny stages.

Weibull cumulative distribution function (Weibull CDF) is one of the most widely used fitting formulas for the curve analysis (Adnadević and Baroš, 2013; Adams et al., 2017; Wason et al., 2018; Yin et al., 2018). The three key points for the vulnerability curves are the best traits to express the embolism resistance and to determine the hydraulic status of the trees. However, the calculated results are not always consistent with the experimental results mentioned above. On the one hand, different tree population species and ontogeny may have various key points (Hammond et al., 2019); hence, we cannot predict all the possible situations with a fixed value. On the other hand, these hydraulic traits are calculated by the “turning melody into straightness” method (Wang and Jiang, 2014) for convenience. Moreover, Domec and Gartner (2001) indicated that Ψ_12_ and Ψ_88_ are only linear approximations of Ψ_e_ and Ψ_l_, respectively.

Based on previous researches and vulnerability curves, the definitions and geometric meanings of the three key points have been clarified as follows. At the “inflection point,” Ψ_m_, the *LC* decreases the fastest, and the curve slope is the largest. Meanwhile, the points Ψ_e_ and Ψ_l_ represent the lower and upper “turning points” of the curve, respectively (Sperry et al., 1988; Pammenter and van der Willigen, 1998; Choat et al., 2012; Torres-Ruiz et al., 2017). With the improvement and popularization of computer technology, including the development and dissemination of scientific computing software, more accurate measurement and calculation methods need to be identified. These methods can be used to determine the three key parameters for the HVC.

*Robinia pseudoacacia* L. is the dominant species in the warm temperate zone (Wang et al., 2020), and it is an anisohydric species, which is sensitive to drought. In addition, it will have a separatrix on the stem when severe drought occurs (Li et al., 2019, 2020), which could provide a suitable opportunity to study the key hydraulic points using experimental methods. This research is first based on the definition and geometric meanings of the three key points, and it combines the hydraulic vulnerability with advanced mathematics. This investigation proposes a differential method (DM) to obtain the precise values of the three key points. Subsequently, we conducted a drought-rewatering experiment on *R. pseudoacacia* by testing the hydraulic status in different drought and rewatering periods to explore the three key points: Ψ_e_, Ψ_m_, and Ψ_l_. With the experimental results, we calculated the three key points by applying the DM and traditional method (TM). We hypothesized that the key points calculated from the DM are more representative of the experimental results.

## Materials and Methods

### Plant Materials

This research was conducted at the Fanggan Research Station at Shandong University in Jinan, Shandong Province, China (36°26′ N, 117°27′ E). The common garden of the station has a mean annual precipitation of 700 ± 100 mm and an average temperature of 13 ± 1°C. Seeds from *Robinia pseudoacacia* L. were collected from a tree in our common garden, and they were stored at 4°C in a refrigerator. These seeds were germinated in a growth chamber in early April 2018. When most seedlings reached 10 cm, healthy and uniform germinants were sown in plastic pots (32 × 29 cm, height × diameter) with an 8 kg mixed sandy loam and humus soil, the soil water holding capacity at full saturation was *c*. 2 kg, and they were allowed to grow for 4 months.

### Experimental Design

In this investigation, 180 well-watered and vigorous saplings that were 4 months old with a similar size were selected for the drought-rewatering experiment. Totally, there were nine treatments or periods. At the beginning of the experiment, for the control group (CK), we randomly selected 20 saplings, 10 of which were for the HVC and stem-specific hydraulic conductivity (*K*_s_) measurement, while the rest were for measuring the stem water potential (Ψ). The remaining 160 saplings that received the drought treatment had their water withheld. We distinguished drought stress by canopy color (Hartmann et al., 2018; Hammond et al., 2019). D_3_ is the mild drought group. Three days after the drought treatment, the leaves began to wilt but were still green. Thereafter, we randomly selected 20 saplings, 10 of which were for the *K*_s_ and maximum stem-specific hydraulic conductivity (*K*_m_) measurement, while the remaining were for measuring the Ψ. D_8_ is the moderate drought group. Eight days after the drought treatment, its leaves wilted and began to turn yellow, and some of the leaf rachis drooped. Further, we randomly selected 20 saplings again, 10 of which were for the *K*_s_ and *K*_m_ measurement, while the others for measuring the Ψ. D_12_ is the severe drought group. After 12 days of receiving the drought treatment, the leaf rachis drooped and became withered, and there was a separatrix on the stem. We then randomly selected 20 saplings, and each sapling was separated from the separatrix into two parts: the upper part (D_12_U) and the lower part (D_12_L), each part was used for the measurement, respectively; 10 saplings were for the *K*_s_ and *K*_m_ measurement, while the others were for measuring the Ψ. Finally, the remaining 100 saplings received continuous rewatering treatment. They were distinguished according to the length of the rewatering time. R_2_ is 2 days after rewatering, R_5_ represents 5 days after rewatering, R_10_ indicates 10 days after rewatering, RR signifies that rewatering occurred until rebudding was present, and RE means that rewatering occurred until new leaves developed, reaching the end of the experiment. All saplings of the rewatering treatments were separated from the separatrix into two parts: the upper part (R_2_U to REU) and lower part (R_2_L to REL), each part was used for the measurement, respectively. When the rewatering days were reached, we randomly selected 20 saplings, 10 of which were for the *K*_s_ and *K*_m_ measurement, and the remaining were for measuring the Ψ. In addition, the leaf area (*LA*), transpiration rate (*E*), and soil water potential (Ψ_s_) were measured for the CK, D_3_, D_8_, and D_12_ treatments. Some key visible treatments are shown in Supplementary Figure 1.

### Transpiration Rate and Leaf Area

The transpiration rate (*E*, mol H_2_O m^−2^ s^−1^) was measured for each sampling day. The fully expanded mature leaves (one leaf per sapling, 10 saplings per treatment) were measured *in situ* using an infrared gas analysis system (Li-6800, Li-Cor, Lincoln, NE, USA). The measurements were conducted at 1,000 μmol m^−2^ s^−1^ photosynthetic photo flux density (PPFD), which was supplied by an external light emitting diode (LED) light. The transpiration rate was measured between 9:00 and 11:00 on sunny days. During the measurement, the temperature, relative humidity, and CO_2_ concentration inside the chamber were controlled at 28°C, 50%, and 400 ppm, respectively. All blades of the leaflets were scanned, and the images were analyzed using the commercial software WinFOLIA Pro 2009a (Regent Instruments, Inc., Quebec, QC, Canada) to determine the leaf area (*LA*, m^2^).

### Stem-Specific Hydraulic Conductivity

The samples were immersed into degassed water as soon as they were cut from the bottom of the stem. Subsequently, the samples were transported promptly to the laboratory with the crowns covered with black plastic bags. All the leaves and bark were removed, and the stems of D_12_ and R_2_ to RE were separated from the separatrix into two parts under water; each segment was 30 cm long. The segments were connected to a hydraulic conductivity measurement system that contained degassed, filtered 20.0 mmol L^−1^ KCl solution. A 30 cm hydraulic head generated hydrostatic pressure to impel water through the segments. The *K*_s_ (kg m^−1^ s^−1^ MPa^−1^) was calculated as follows:

(1) $$\begin{array}{l} K_{\text{s}}=\frac{LQ_{\text{m}}}{Ap} \end{array}$$

(2) $$\begin{array}{l} Q_{\text{m}}=\frac{m}{t} \end{array}$$

where *L, Q*_m_, *A, p, m*, and *t* represent the length of the segment (m), mass of water per unit of time through a segment (kg s^−1^), average cross-sectional area for both ends of the stem (m^2^), intensity of the water pressure across the segment (MPa), mass of water through the segment (kg), and time for the conductance measurement (s), respectively. Then, *K*_m_ (kg m^−1^ s^−1^ MPa^−1^) was measured after the segment was flushed for 30 min with degassed, filtered 20.0 mmol L^−1^ KCl solution under 0.10 MPa pressure to remove any air bubbles in the xylem.

### Water Potential

The stem water potential (Ψ, –MPa) was measured in a pressure chamber (1505D-EXP; PMS Instrument Company, Albany, OR, USA). Ten samples for each treatment were collected simultaneously between 9:00 and 11:00, at the same time when the other 10 samples for the *K*_s_ measurements were cut down. Samples were cut from the saplings, sealed in plastic bags containing moist paper towels, and stored in a cooler before the stem water potentials were measured in a laboratory near the common garden. In addition, the soil water potential (Ψ_s_, –MPa) was measured using the same repetition as stem water potential with a dew point hygrometer (WP4C, decagon devices, München, Germany), the temperature in sample room was set at 25.0°C, we found that fine root mostly concentrated at the lower part of the pot, therefore soil samples were collected at *c*. 5 cm higher than the bottom center of the pot.

### Loss of Conductivity at Different Pressures

After the *K*_m_ measurement, the segments were fixed in double-sleeved air-injection chambers (1505D-EXP, PMS Instrument Co, Albany, OR, USA). *K*_s_ was then measured after exposing the segments to progressively increased air-injection pressures that range from 0.00 to 4.00 MPa, at 0.20 MPa steps, and then 4.50, 5.00, and 6.00 MPa, according to the characteristics of the curve and our previous research (Liu et al., 2020). The air-injection pressure remained constant at each injection pressure level using a gas pressure regulator for 5 min. After the pressure was released, the injected samples were allowed to achieve equilibration over 10 min until no bubbles were discharged from the xylem. After this period, the post-injection *K*_s_ was determined. The *LC* after the air injection at each pressure level was calculated as follows:

(3) $$\begin{array}{l} LC_{i}=\frac{K_{\text{m}}-K_{\text{si}}}{K_{\text{m}}} \end{array}$$

where *i* is the times of air-injection (from 0 to 23). For convenience of calculation, we combined Equation (3) with Equations (1) and (2) to derive Equation (4).

(4) $$\begin{array}{l} LC_{i}=1-\frac{T}{t_{i}} \end{array}$$

where *T* denotes the time of the water conductance at the *K*_m_ (s).

### Xylem Water Gain and Loss Estimation

In this research, we neglected the effect of the shoot surface (foliar) water uptake and stem evaporation on the xylem water gain (*WG*, kg s^−1^) and water loss (*WL*, kg s^−1^), although they may have physiological significance (Fuenzalida et al., 2019; Schreel and Steppe, 2019). We only calculated the primary factors that affect the water balance of the plants, the amount of water that passes through the xylem per unit of time, the amount of water that is evaporated by all leaves per unit of time, and the difference between them. We did not estimate the xylem water gain and water loss in the rewatering groups as there were no functional leaves in those treatments. They were calculated as follows:

(5) $$\begin{array}{l} WG=K_{\text{m}}\times \Delta \Psi \times A\times \left(1-LC\right)÷L \end{array}$$

(6) $$\begin{array}{l} WL=E\times LA \end{array}$$

where ΔΨ represents the difference between Ψ_s_ and Ψ (MPa). We calculated the net water resource xylem that is gained from the soil as the difference between *WG* and *WL*.

### Curve Fitting and Differential Method Calculation

HVCs were fitted using the Weibull CDF as demonstrated in Equation (7).

(7) $$\begin{array}{l} y=LC=1-\text{exp }\left[-{\left(\frac{\Psi }{a}\right)}^{b}\right],\Psi \in \left[0,6\right] \end{array}$$

where Ψ represents the progressively increased air-injection pressures that the samples were exposed to, and it is the absolute value of stem water potential. In addition, *a* and *b* are constants that match the Weibull CDF. In most cases, *a* satisfies the condition *a* > 0. We calculated the first, second, and third derivatives of the Weibull CDF as follows:

(8) $$\begin{array}{l} y^{\prime }=\frac{b}{a}\text{exp }\left[-{\left(\frac{\Psi }{a}\right)}^{b}\right]{\left(\frac{\Psi }{a}\right)}^{b-1},\Psi \in \left[0,6\right] \end{array}$$

(9) $$\begin{array}{l} y^{\prime \prime }=\frac{b}{a^{2}}\text{exp }\left[-{\left(\frac{\Psi }{a}\right)}^{b}\right]{\left(\frac{\Psi }{a}\right)}^{b-2}\\ \;\left[b-1-b{\left(\frac{\Psi }{a}\right)}^{b}\right],\Psi \in \left[0,6\right] \end{array}$$

(10) $$\begin{array}{l} y^{\prime \prime \prime }=\frac{b^{3}}{a^{3}}\text{exp }\left[-{\left(\frac{\Psi }{a}\right)}^{b}\right]{\left(\frac{\Psi }{a}\right)}^{3b-3}-\frac{3\left(b-1\right)b^{2}}{a^{3}}\\ \text{ exp }\left[-{\left(\frac{\Psi }{a}\right)}^{b}\right]{\left(\frac{\Psi }{a}\right)}^{2b-3}+\frac{b\left(b-1\right)\left(b-2\right)}{a^{3}}\\ \text{ exp }\left[-{\left(\frac{\Psi }{a}\right)}^{b}\right]{\left(\frac{\Psi }{a}\right)}^{b-3},\;\Psi \in \left[0,6\right] \end{array}$$

where *y*′ is the first derivative of the Weibull CDF; ecologically, it is the slope or changing rate of the *LC*. Next, *y*″ is the second derivative of the Weibull CDF, which is the changing rate of the slope. Finally, *y*^‴^ is the third derivative of the Weibull CDF. Based on the definition and geometric meanings of the three key points and combining the hydraulic vulnerability with advanced mathematics, this research proposes a DM to calculate the key points. Ψ_m_ is the inflection point where *y*″ = 0. Ψ_e_ is the lower left turning point when *y*^‴^ = 0, while Ψ_l_ is the upper right turning point when *y*^‴^ = 0.

According to the DM, the three key points were calculated as follows:

(11) $$\begin{array}{l} \Psi_{\text{m}}=a\sqrt[{b}]{\frac{b-1}{b}} \end{array}$$

(12) $$\begin{array}{l} \Psi_{\text{e}}=a\sqrt[{b}]{\frac{3\left(b-1\right)-\sqrt{\left(b-1\right)\left(5b-1\right)}}{2b}} \end{array}$$

(13) $$\begin{array}{l} \Psi_{\text{l}}=a\sqrt[{b}]{\frac{3\left(b-1\right)+\sqrt{\left(b-1\right)\left(5b-1\right)}}{2b}} \end{array}$$

The corresponding *LC* was then calculated as *LC*_e_, *LC*_m_, and *LC*_l_. We also calculated Ψ_12_, Ψ_50_, and Ψ_88_ through the TM.

(14) $$\begin{array}{l} \Psi_{12}=a\sqrt[{b}]{2\log_{\text{e}}5-{\text{log}}_{\text{e}}22} \end{array}$$

(15) $$\begin{array}{l} \Psi_{50}=a\sqrt[{b}]{\log_{e}2} \end{array}$$

(16) $$\begin{array}{l} \Psi_{88}=a\sqrt[{b}]{2\log_{e}5-\log_{e}3} \end{array}$$

### Statistics

The data were first tested for normality and homogeneity. One-way analysis of variance (ANOVA) was used to identify the differences among all the treatments. All ANOVAs were followed by Duncan (for homogeneity) or Tamhane (for heterogeneity) multiple comparison tests, which were performed at α = 0.05, and significant differences were found. One sample *t*-test was used to determine if the calculating results can represent the experimental results. Linear regression was used to determine the relationship between Ψ and Ψ_s_, and between *E* and *K*_s_. The data analysis was performed using SPSS 26 (SPSS Inc., Chicago, IL, USA). The derivatives were obtained by MATLAB 2016a (MathWorks Inc., Natick, Massachusetts, USA). The curve fittings and all figures were drawn using Origin 2019b (Originlab Co., Northampton, MA, USA).

## Results

**Figure 1:**
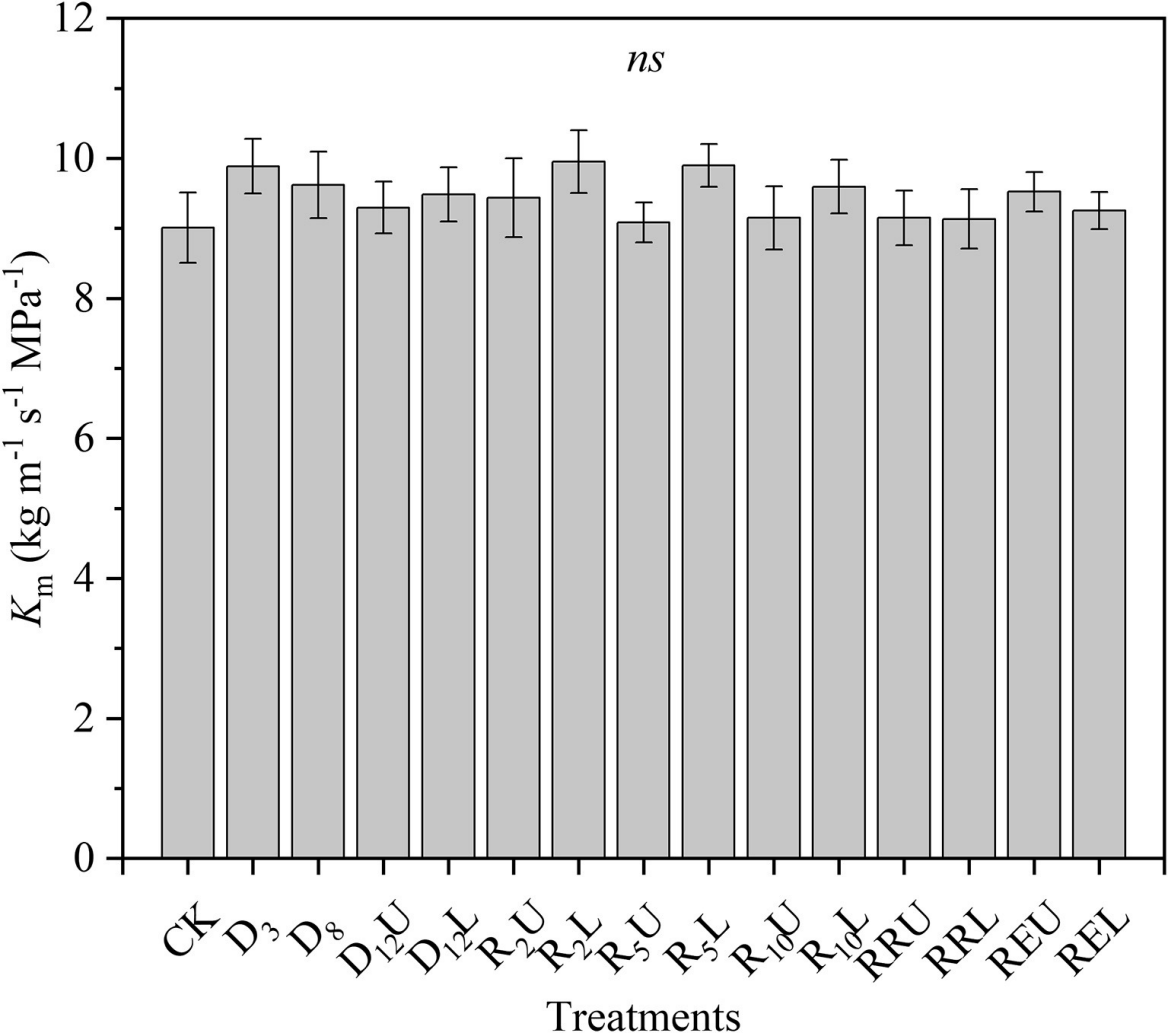
Maximum stem-specific hydraulic conductivity (*K*_m_) for all treatments. CK, control group; D_3_, mild drought group; D_8_, moderate drought group; D_12_U, upper part of the severe drought group; D_12_L, lower part of the severe drought group; R_2_U, upper part of the 2-day-rewatering group; R_2_L, lower part of the 2-day-rewatering group; R_5_U, upper part of the 5-day-rewatering group; R_5_L, lower part of the 5-day-rewatering group; R_10_U, upper part of the 10-day-rewatering group; R_10_L, lower part of the 10-day-rewatering group; RRU, upper part of the group, in which rewatering occurred until rebudding was present; RRL, lower part of the group, in which rewatering occurred until rebudding was present; REU, upper part of the rewatering group to the end of the experiment; REL, lower part of the rewatering group to the end of the experiment. The data is represented by the mean ± 1 *SE* and *n* = 10. One-way ANOVA and Duncan multiple comparisons were performed to detect the differences among all the treatments; *ns* indicates no significant difference.

There were no significant differences among the *K*_m_ for all treatments; the means of all treatments ranged from 9.008 to 9.952 kg m^−1^ s^−1^ MPa^−1^ (Figure 1).

The Weibull CDF accurately fit the HVC according to the coefficients of determination (*R*^2^ = 0.999, *P* < 0.01). The result of the fitting is as follows:

(17) $$\begin{array}{l} LC=1-\text{exp }\left[-{\left(\frac{\Psi }{2.23}\right)}^{2.50}\right],\Psi \in \left[0,6\right] \end{array}$$

**Figure 2:**
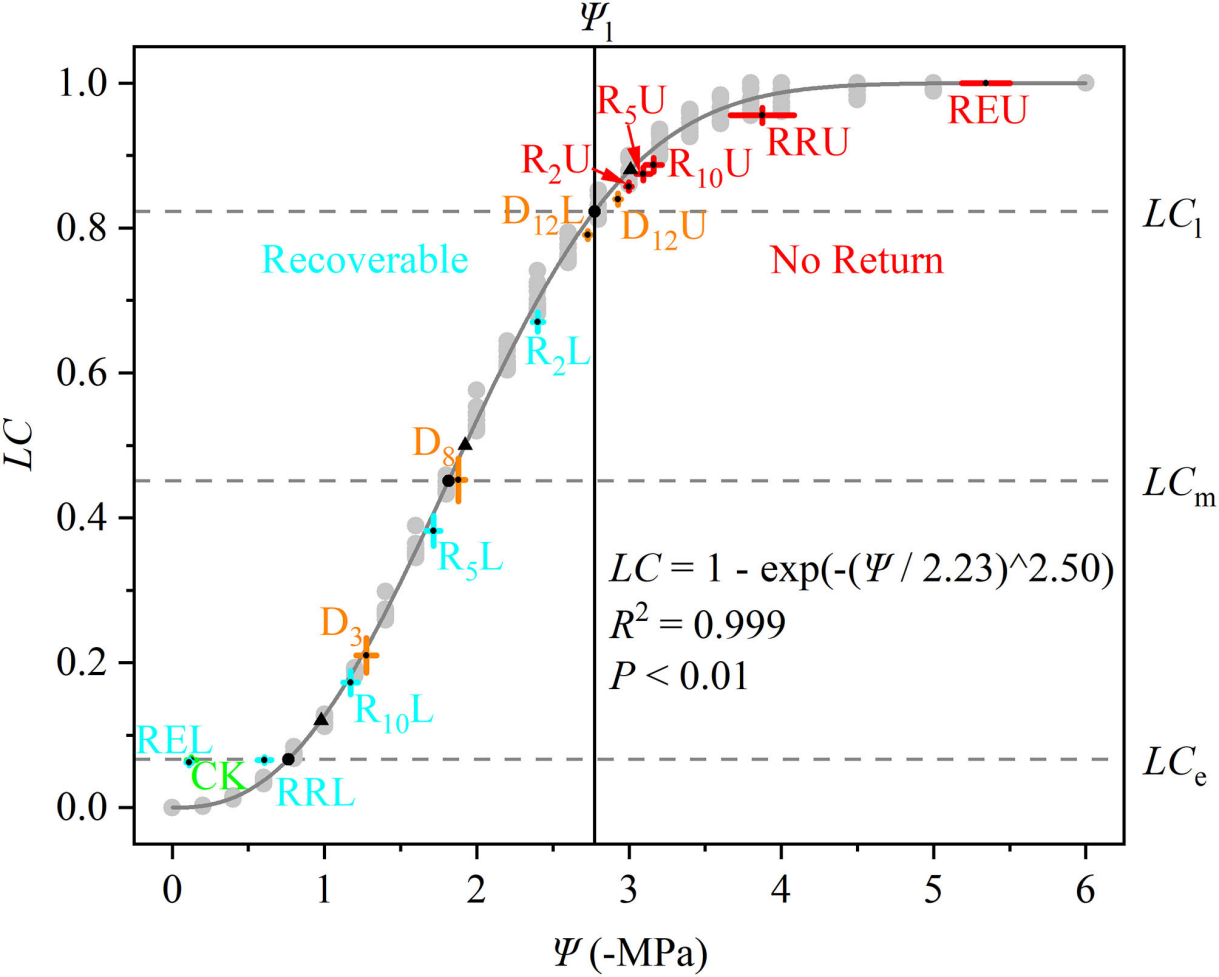
Hydraulic vulnerability curve (gray solid line) for *Robinia pseudoacacia*, which is fitted from 10 saplings belonging to CK (light gray points). The stem water potential (Ψ, –MPa) and loss of conductivity (*LC*) for all of the treatments are marked in the figure. The data is represented by the mean ± 1 *SE* and *n* = 10. CK, control group; D_3_, mild drought group; D_8_, moderate drought group; D_12_U, upper part of the severe drought group; D_12_L, lower part of the severe drought group; R_2_U, upper part of the 2-day-rewatering group; R_2_L, lower part of the 2-day-rewatering group; R_5_U, upper part of the 5-day-rewatering group; R_5_L, lower part of the 5-day-rewatering group; R_10_U, upper part of the 10-day-rewatering group; R_10_L, lower part of the 10-day-rewatering group; RRU, upper part of the group in which rewatering occurred until rebudding was present; RRL, lower part of the group in which rewatering occurred until rebudding was present; REU, upper part of the group in which rewatering occurred until the end of the experiment; REL, lower part of the group in which rewatering occurred until the end of the experiment. Ψ_e_, Ψ_m_, and Ψ_l_ are bottom up in the black circles. In addition, *LC*_e_, *LC*_m_, and *LC*_l_ (gray dash lines) are the corresponding *LC* of the Ψ_e_, Ψ_m_, and Ψ_l_. Black triangles indicate Ψ_12_, Ψ_50_, and Ψ_88_. Ψ at Ψ_l_ (black vertical solid line) separates the curve into two parts; the left part is recoverable, while the right part cannot be recovered.

D_12_U and R_2_U to REU were along the right side of Ψ_l_ (no return zone), while the other treatments were along the left side of Ψ_l_ (recoverable zone). The *LC* of CK, RRL, and REL were similar to *LC*_e_, while D_8_ was close to Ψ_m_, and D_12_L and D_12_U were on both sides of Ψ_l_ (Figure 2).

**Figure 3:**
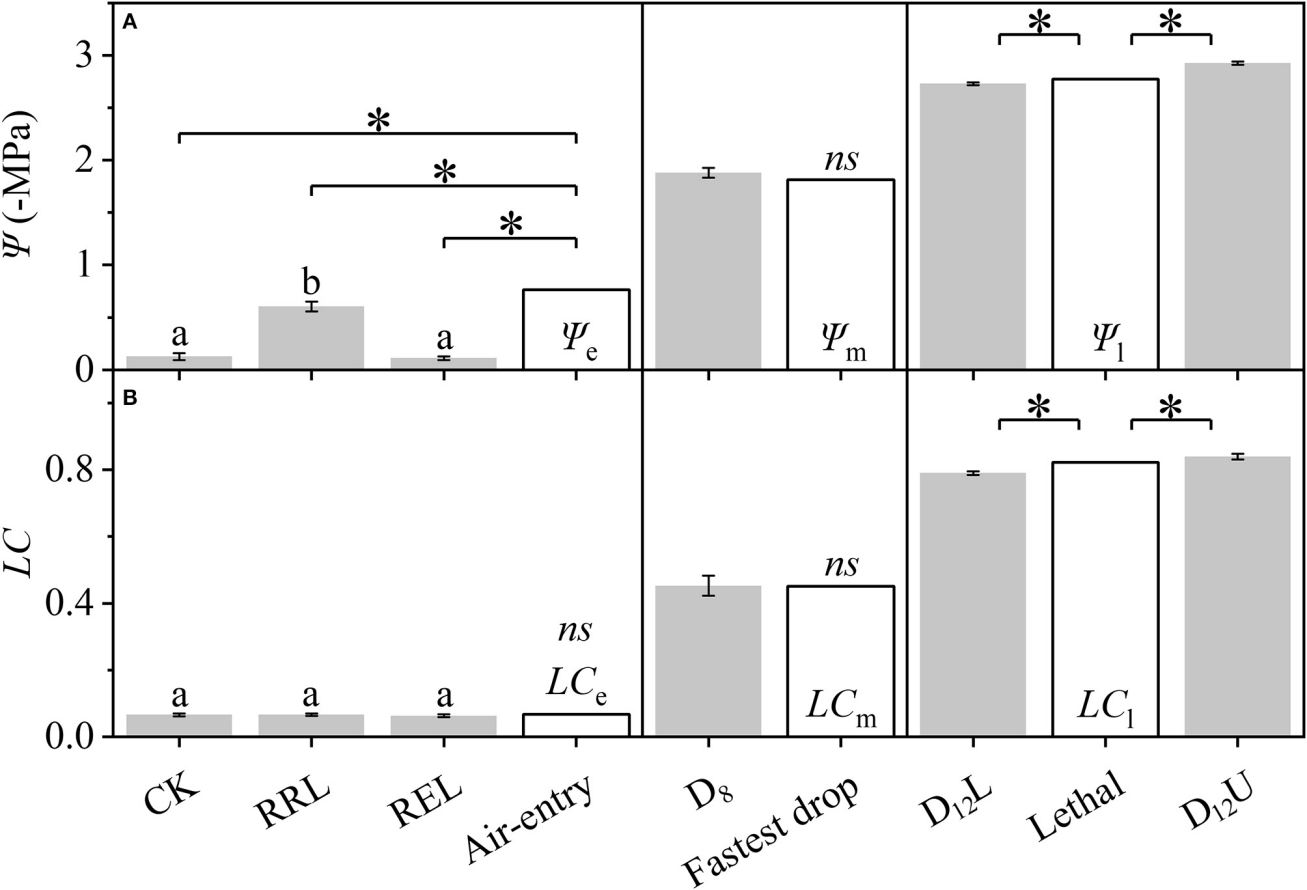
Differences among the CK, RRL, REL, and Ψ_e_, between D_8_ and Ψ_m_, and among D_12_L, Ψ_l_, and D_12_U for the stem water potential (Ψ, **A**) and loss of conductivity (*LC*, **B**). CK, control group; D_8_, moderate drought group; D_12_U, upper part of the severe drought group; D_12_L, lower part of the severe drought group; RRL, lower part of the group in which rewatering occurred until rebudding was present; REL, lower part of the group in which rewatering occurred until the end of the experiment. The data that belongs to CK, RRL, REL, D_8_, D_12_L, and D_12_U are represented by the mean ± 1 *SE* and *n* = 10. Data of the three key points are signified by the calculated results via the DM. One-way ANOVA and Duncan multiple comparisons were performed to detect the differences among CK, RRL, and REL; different letters indicate significant differences where *P* < 0.05. The one-sample *t*-test was used to detect the difference between the calculated and experimental results; *ns* indicates no significant difference; the symbol *P < 0.05.

Based on Figure 2, the differences among the CK, RRL, REL, and Ψ_e_, between D_8_ and Ψ_m_, among D_12_L, D_12_U, and Ψ_l_ (Figure 3), were further examined. We tested that CK and REL do not have a noticeable difference; however, CK and REL have significant differences with RRL in Ψ. In addition, CK, RRL, and REL are significantly smaller in Ψ than Ψ_e_. Meanwhile, for the *LC* of CK, RRL, and REL, there is a noticeable difference with *LC*_e_. Ψ and *LC* of D_8_ are equal to Ψ_m_ and *LC*_m_, respectively. Ψ_l_ and *LC*_l_ are significantly larger than Ψ and *LC* of D_12_L, although they are significantly smaller than those of D_12_U, respectively.

**Figure 4:**
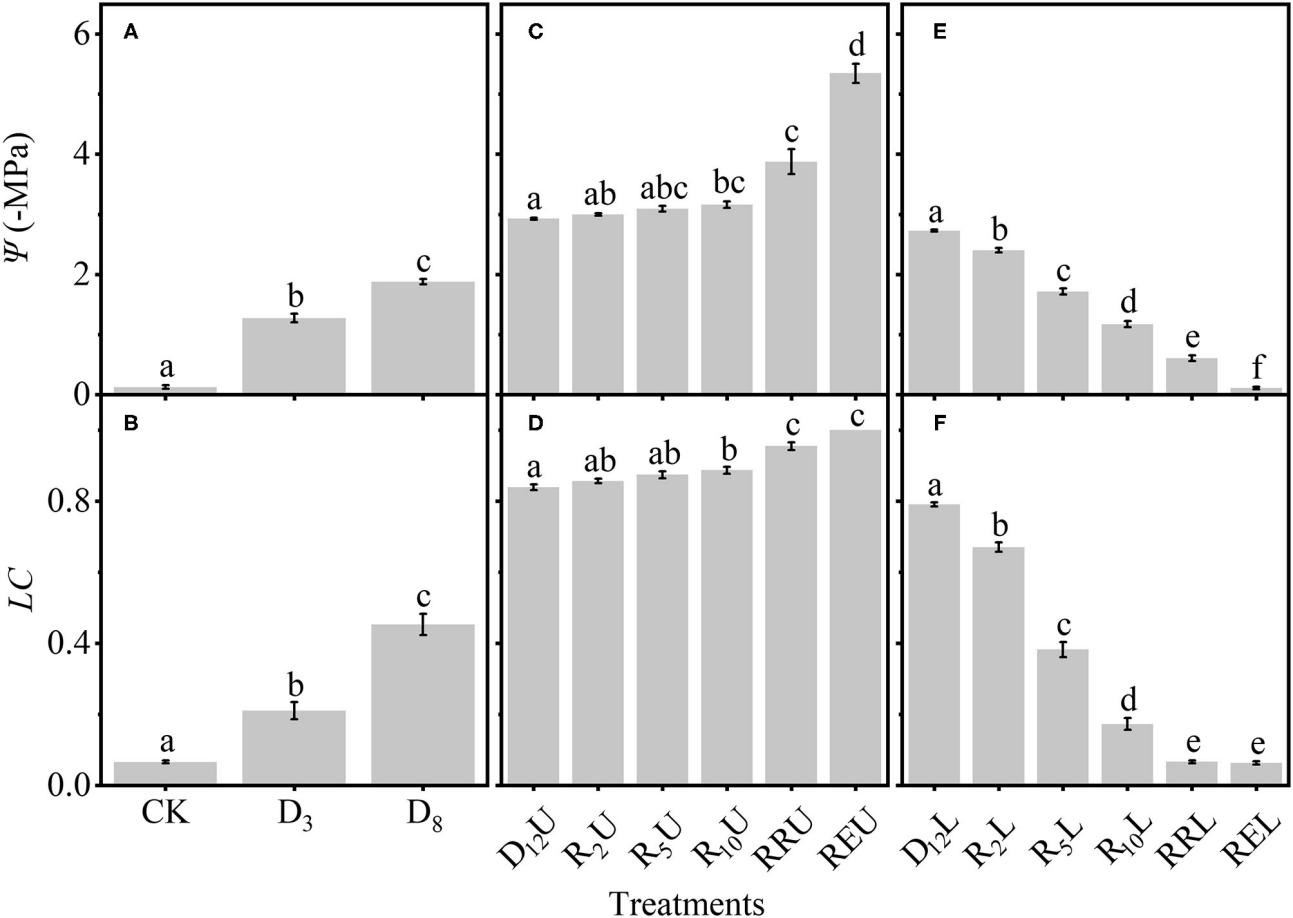
Differences among: CK, D_3_, D_8_ **(A)**; D_12_U, R_2_U to REU **(C)**; D_12_L, R_2_L to REL **(E)** for the stem water potential (Ψ). The loss of conductivity (*LC*) differences among CK, D_3_, D_8_ **(B)**; D_12_U, R_2_U to REU **(D)**; and D_12_L, R_2_L to REL **(F)**. CK, control group; D_3_, mild drought group; D_8_, moderate drought group; D_12_U, upper part of the severe drought group; D_12_L, lower part of the severe drought group; R_2_U, upper part of the 2-day-rewatering group; R_2_L, lower part of the 2-day-rewatering group; R_5_U, upper part of the 5-day-rewatering group; R_5_L, lower part of the 5-day-rewatering group; R_10_U, upper part of the 10-day-rewatering group; R_10_L, lower part of the 10-day-rewatering group; RRU, upper part of the group, in which rewatering occurred until rebudding was present; RRL, lower part of the group, in which rewatering occurred until rebudding was present; REU, upper part of the group, in which rewatering occurred until the end of the experiment; REL, lower part of the group, in which rewatering occurred until the end of the experiment. The data is represented by the mean ± 1 *SE*, and *n* = 10. One-way ANOVA and Tamhane multiple comparisons were performed to detect the differences. In addition, different letters indicate significant differences, where *P* < 0.05.

Subsequently, we tested the differences among the treatments (Figure 4). To make the results more intuitive and scientific, we separated the treatments into three groups. Figures 4A,B depict that Ψ and *LC* significantly increased for CK, D_3_, and D_8_ by increasing the drought stress. Figures 4C,D demonstrate that for D_12_U and R_2_U to REU, by increasing the rewatering time, there is no apparent change for Ψ and *LC* (D_12_U to R_10_U); then, Ψ and *LC* increased to a high level (R_10_U to REU). However, by further increasing the rewatering time, Ψ and *LC* of D_12_L and R_2_L to REL decreased significantly (Figures 4E,F).

**Figure 5:**
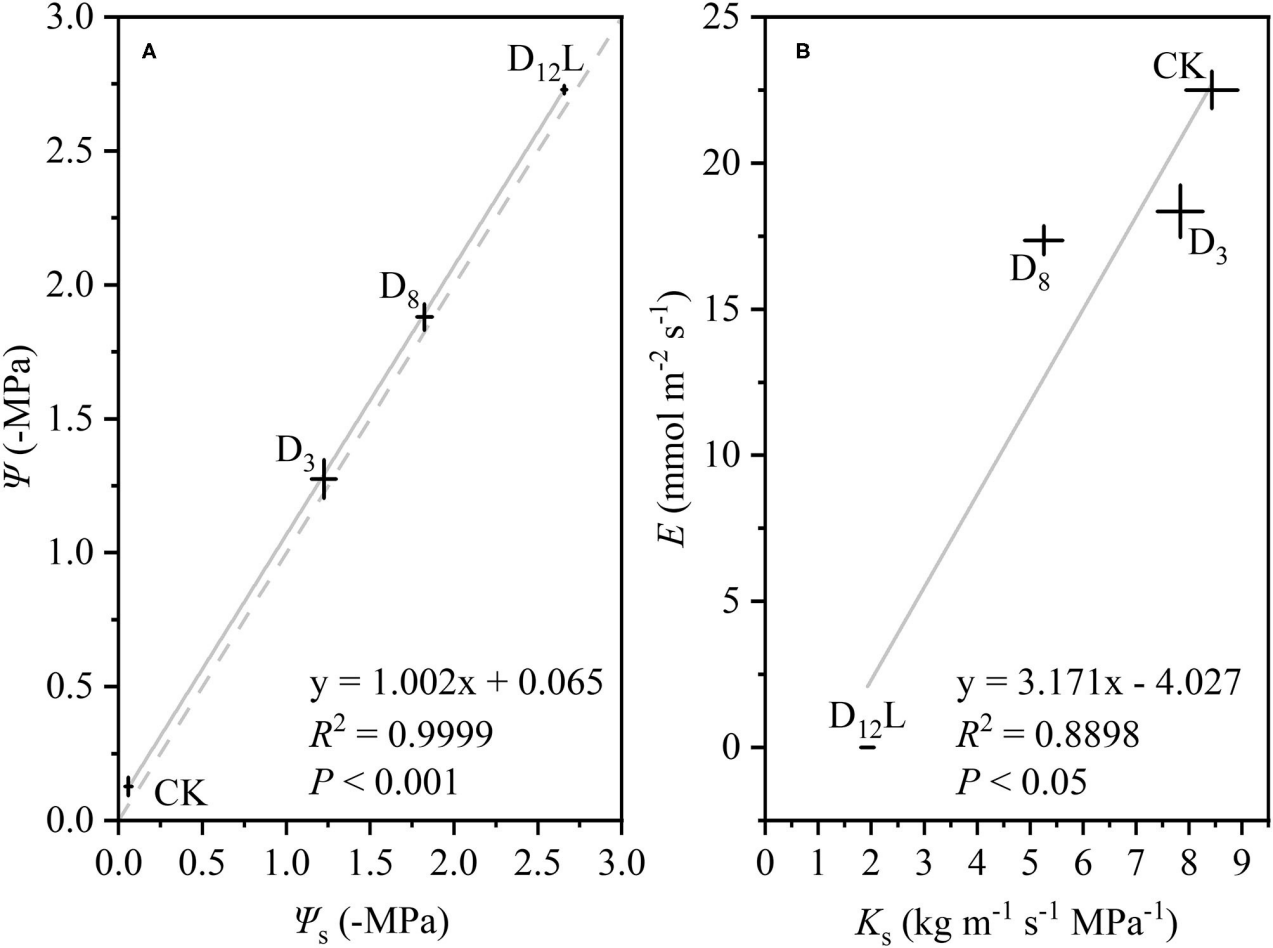
The relationship between soil water potential (Ψ_s_, –MPa) and stem water potential (Ψ, –MPa), **(A)** between stem-specific hydraulic conductivity (*K*_s_, *k*_g_ m^−1^ s^−1^ MPa^−1^) and transpiration rate (*E*, mol m^−2^ s^−1^), **(B)** in CK, D_3_, D_8_, and D_12_L. CK, control group; D_3_, mild drought group; D_8_, moderate drought group; D_12_L, lower part of the severe drought group. The data is represented by the mean ± 1 SE and *n* = 10. Light gray line stands for the linear regression of the points, and light gray dash line stands for Ψ = Ψ_s_.

**Figure 6:**
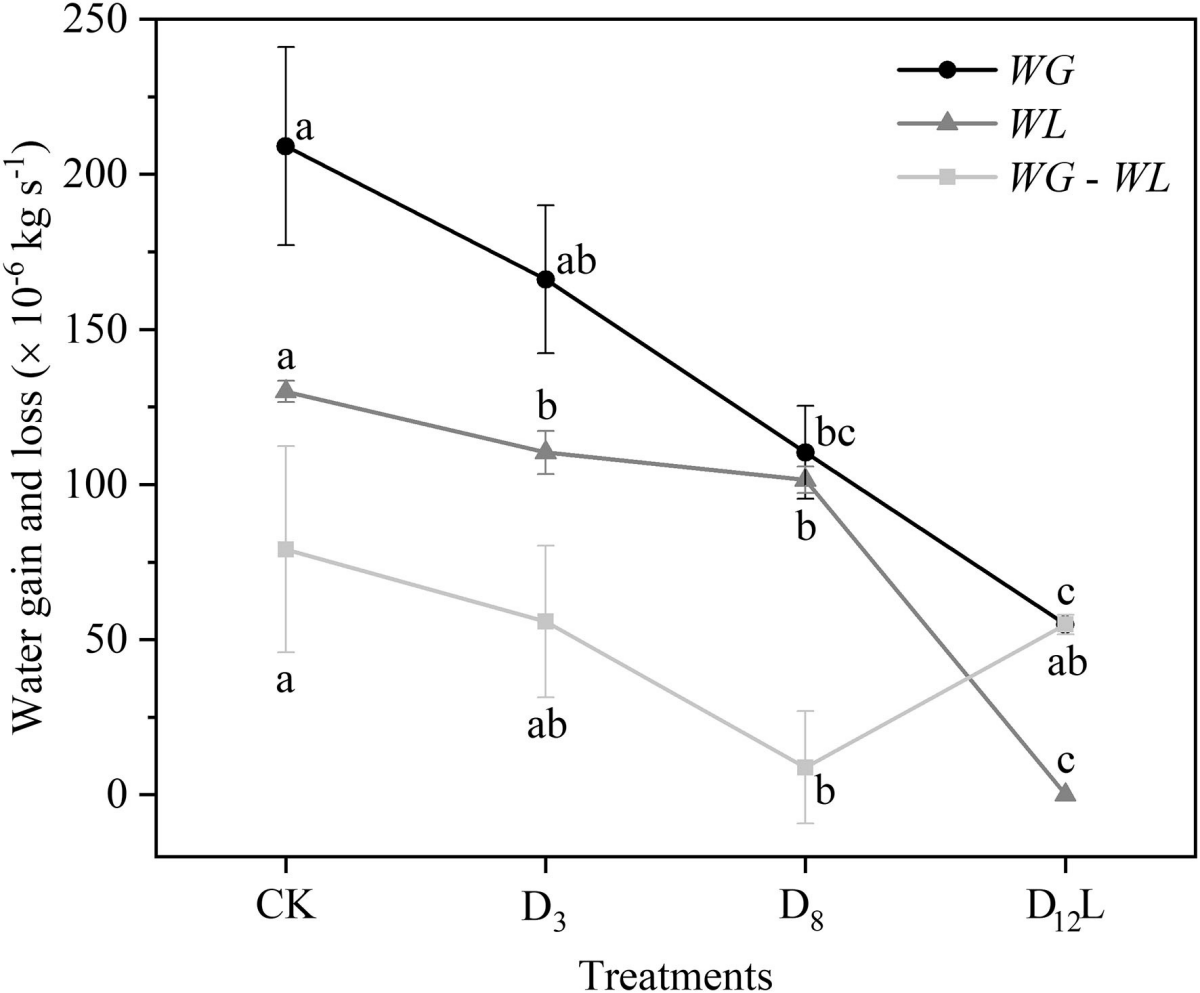
Water gain (*WG*) and water loss (*WL*) of CK, D_3_, D_8_, and D_12_. CK, control group; D_3_, mild drought group; D_8_, moderate drought group; D_12_L, lower part of the severe drought group. Black circles represent *WG*, gray triangles indicate *WL*, and light gray squares represent the difference between *WG* and WL. The data is represented by the mean ± 1 *SE* and *n* = 10. One-way ANOVA and Duncan multiple comparisons were performed to detect the differences among *WG* and *WL* and *WG* – *WL* in CK, D_3_, D_8_, and D_12_L. In addition, different letters indicate significant differences, where *P* < 0.05.

With the increase in the drought stress (Ψ_s_), Ψ increased linearly (*R*^2^ = 0.9999, *P* < 0.001; Figure 5A), K_s_ and E decreased linearly (*R*^2^ = 0.8898, *P* < 0.05; Figure 5B). *WG* and *WL* decreased significantly, while the difference between *WG* and *WL* reached the minimum value at D_8_ (Figure 6).

**Figure 7:**
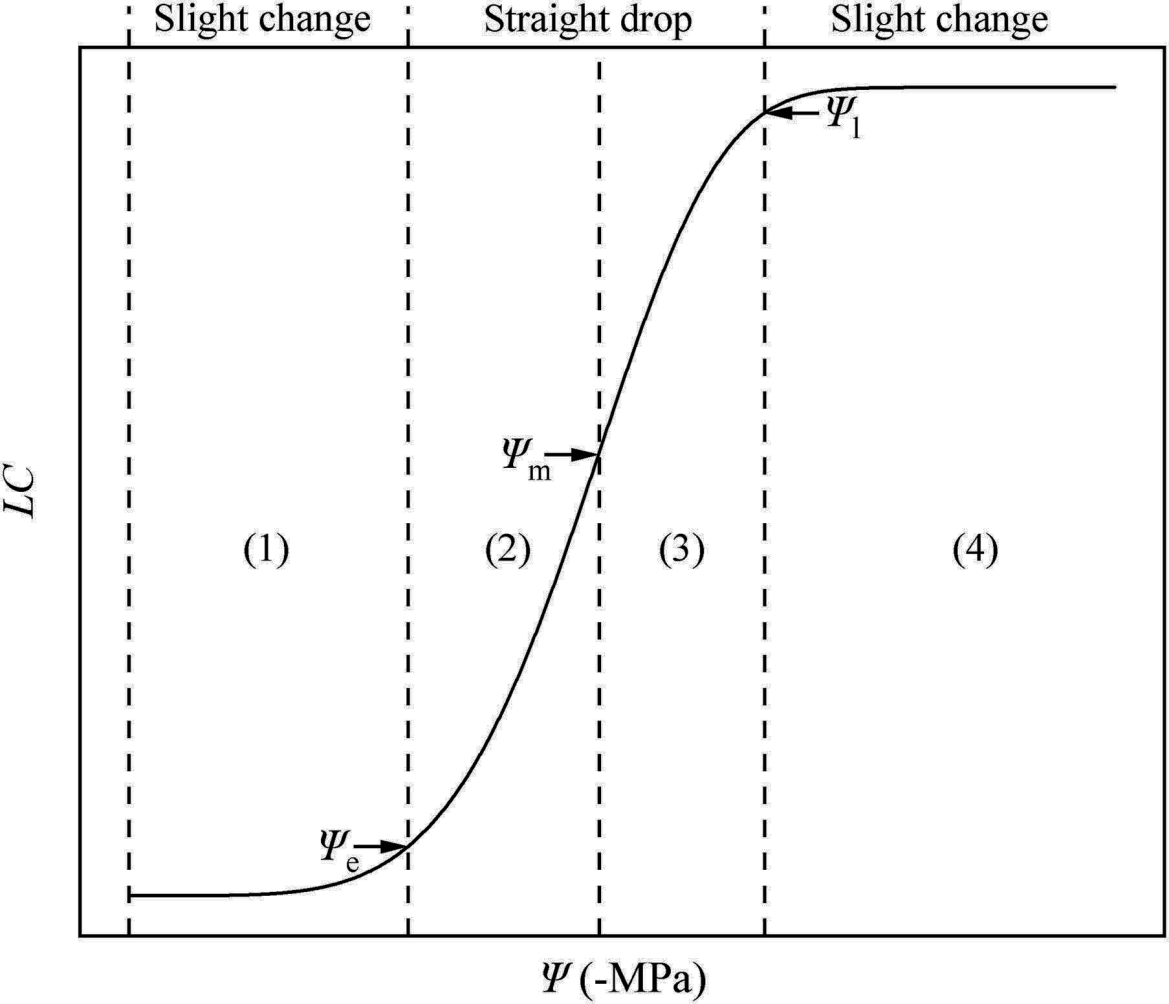
Hydraulic vulnerability curve diagram. The water potential is represented by Ψ, and the loss of conductivity is represented by *LC*. There are four periods in the HVC: (1) stationary period, (2) accelerated decline period, (3) decelerated decline period, (4) and platform period. These are separated by three key points: air-entry point (Ψ_e_), fastest drop point (Ψ_m_), and upper inflection point (Ψ_l_).

Using the key points calculated using the DM, we divided the HVC into four parts (Figure 7). In parts (1) and (4), when the water potential becomes larger, the change of hydraulic conductivity is less than that in parts (2) and (3). In other words, a slight change in (1) and (4) is observed, while a straight drop is observed in (2) and (3).

The visible periods included D_8_, D_12_, RR, and RE. The separatrix, new buds, and new leaves are clearly visible in Supplementary Figure 1.

## Discussion

### Drought Did Not Change the Water Transport Efficiency

There was no significant difference in the *K*_m_ for all the treatments in this research (Figure 1). This indicates that during the experiment, the xylem structure of *R. pseudoacacia* did not have a noticeable change (Choat et al., 2012), and the differences of the xylem function were completely caused by the treatments. However, our treatments did not change the water transport efficiency (Figure 1), according to the xylem efficiency-safety tradeoff, which meant that a balance existed between hydraulic efficiency and safety (Gleason et al., 2016; Liu et al., 2019). It can be concluded that the water transport safety of *R. pseudoacacia* has not changed significantly during the experiment; thus, we can only use one HVC to examine the hydraulic vulnerability for all the treatments. Notably, some researches indicated that the “air-injection” method may be prone to artifacts if the maximum length of the xylem vessels is not considered when preparing the samples for conducting the measurements (Ennajeh et al., 2011). However, this research was based on 4-month-old saplings, it is impossible to have a long conduit like a tree, according to the shape of our HVC (Figure 2) and previous researches (Zhu et al., 2018; Li et al., 2019, 2020; Liu et al., 2020), we convince that the 30-cm-long segments had no open vessels, so that the “air-injection” method did not cause experimental artifacts in this research. Moreover, the “air-injection” method can accurately control the stem water potential, and it can improve the precision of the HVC (Sergent et al., 2020).

### Calculated Result From Differential Method Can Better Estimate the Experimental Results

With the rise in the drought stress, Ψ and *LC* of *R. pseudoacacia* increased (Figures 2, 4A,B). After rewatering, Ψ and *LC* of the stem above the separatrix did not recover. However, Ψ and *LC* were maintained at the initial level from D_12_U to R_10_U, after which Ψ and *LC* increased significantly, and then achieved full embolism (Figures 4C,D). In addition, the stem below the separatrix began to recover (Figures 4E,F). According to the hydraulic segmentation hypothesis, plants maintained the hydraulic status of the stems by reducing the transpiration through defoliation; thus, Ψ and *LC* of xylem exhibited no apparent change. The question arises to why D_12_L can recover from the drought stress after rewatering whereas D_12_U cannot. It is possible that the water resource of D_12_U can get through the hydraulic conductance. However, this would never meet their metabolic needs, let alone rebudding, even if they were rewatered, in which they “passed the point of no return.” In contrast, the water resource of D_12_L that was gained from the hydraulic conductance achieved their metabolic needs (the value was ~54.89 × 10^−6^ kg s^−1^, Figure 6). After recovery, they can rebud. By comparing these two parts (Figure 2), we determined that, although their Ψ and *LC* are close, their responses after rewatering were inconsistent. Like the “squeeze theorem” (Wang and Jiang, 2014), the lethal point of *R. pseudoacacia* was at a point that ranged from 2.73 to 2.93 MPa, and the corresponding *LC* ranged from 0.79 to 0.84 (Figure 3). Meanwhile, Ψ_l_ (=2.77 MPa) that was obtained by the DM was between 2.73 and 2.93, and *LC*_l_ (=0.82) ranged from 0.79 to 0.84. Therefore, we demonstrated that Ψ of D_12_L < Ψ_l_ < Ψ of D_12_U (*P* < 0.05) and *LC* of D_12_L < *LC*_l_ < *LC* of D_12_U (*P* < 0.05). In addition, Ψ_88_ overestimated the lethal point (Figure 3). Based on our experimental result, by combining the definition and geometric meanings of the lethal point, we recommend that Ψ_l_, which is obtained by the DM, is the lethal point (the point of no return) of *R*. *pseudoacacia*.

We tested that the *LC* of CK, RRL, and REL were concentrated next to *LC*_e_ (Figure 3B); however, their Ψ values were significantly smaller than Ψ_e_. This indicated that when *LC* was reduced to the control level, although Ψ continued to decrease, the *LC* would never be reduced but maintained at a certain level. In other words, the *K*_s_ for CK and REL were still lower than *K*_m_. This may be because *R*. *pseudoacacia* has some natural embolism that was not induced by stress (Li et al., 2020). Consequently, the actual *K*_m_ is smaller than the theoretical *K*_m_. Natural embolism may exist because *R*. *pseudoacacia* is anisohydric, and its Ψ and *LC* changes with the changing environment (Li et al., 2019). Moreover, recovery of natural embolism would consume a significant amount of resources; however, it would produce less benefits, which goes against the resource trade-off theory. Conversely, when facing drought stress, at the period when Ψ is raised from 0 to Ψ_e_, because of natural embolism, the *K*_s_ would never decline significantly. Nevertheless, when Ψ > Ψ_e_, *K*_s_ starts to decrease. This conforms with our definition of the air-entry point. Therefore, Ψ_e_ is possibly the ultimate Ψ that can enable the plant to maintain the actual *K*_m_. Consequently, we recommend to have Ψ_e_ as the air-entry point of *R*. *pseudoacacia*, which is obtained by the DM.

In addition, we observed that Ψ and Ψ_m_ as well as *LC* and *LC*_m_ have no noticeable difference at D_8_ (Figure 3). It was hypothesized that under the increasing drought stress (Figure 5A), *K*_s_ and *E* decreased linearly (Figure 5B), *WG* and *WL* decreased. However, the difference between *WG* and *WL* reached the minimum value at D_8_ (Figure 6). At that point, the net water resource xylem was gained from the soil (~8.83 × 10^−6^ kg s^−1^, Figure 6), and its metabolic requirements cannot be satisfied (~54.89 × 10^−6^ kg s^−1^, Figure 6). The plant can only meet the water demand by reducing the water content of xylem, leading to rapid diffusion of embolism, and at that point, *LC* increases the fastest. Therefore, during D_8_-D_12_, the leaves started to dry and fall off. In addition, they form a hydraulic segmentation, which ensures metabolic water at the expense of transpiration, thereby slowing down the increase of the *LC*. Accordingly, we can determine that Ψ_m_ is the fastest drop point of *R*. *pseudoacacia*.

Furthermore, we tested and compared the results obtained by Hammond et al. (2019) with those of the DM, and the results were found to be the same. Therefore, we can conclude that the differences between the experimental and calculated results can be attributed to the linear progressive method of the “turning melody into straightness,” and the DM can eliminate the differences. A significant amount of work is required to perfect this method. As indicated by Hammond et al. (2019), continued experimentation is necessary to assess the different tree species, populations, and individuals in different ontogeny stages.

### Four Periods of HVC for Better Understanding of Hydraulic State

By applying Ψ_e_, Ψ_m_, and Ψ_l_, we can divide the HVC into four periods (Figure 7), including (1) the stationary period (0 ≤ Ψ < Ψ_e_). Currently, the Ψ is low, and the *K*_s_ may be at the theoretical *K*_m_, similar to *P. taeda* (Hammond et al., 2019), or at the actual *K*_m_, similar to *R*. *pseudoacacia*. When the plants are facing drought stress, the absolute value of Ψ increases, whereas *K*_s_ slightly decreases or remains largely unchanged. As Ψ increases to more than Ψ_e_, the plants can no longer maintain the *K*_s_ at the theoretical or actual *K*_m_. (2) From this point forward, the entry of air causes the hydraulic conductivity to decrease linearly. In addition, the stem of the plant enters a period of accelerated decline from the stationary period (Ψ_e_ ≤ Ψ < Ψ_m_), during which the aggravation of stress continues to cause Ψ to increase. In other words, a slight change in Ψ will cause a large drop in the hydraulic conductivity. This is due to the increasing drought stress and the undiminished transpiration of the entire plant. In particular, when Ψ = Ψ_m_, the hydraulic conductivity exhibits the fastest drop rate, after which it proceeds to a period of (3) decelerated decline (Ψ_m_ ≤ Ψ < Ψ_l_). In this period, as mentioned in the hydraulic segmentation hypothesis, the water resource that xylem gained from the soil cannot satisfy the transpiration and metabolic needs; hence, the leaves begin to dry and fall off. To satisfy the stem metabolism and protect the stem from severe embolism, the increase of Ψ and *LC* slows down (before the lethal point). (4) When Ψ_l_ ≤ Ψ, although the branches of the plant do not completely lose their hydraulic conductivity, they lose their ability to recover. At this stage, the stem of the plant enters the platform period until Ψ arrives at the highest point. These four periods belong to the same vulnerability curve due to their different ecological significance and mathematical properties. Our results prove again the significance of the HVC in studying plant responses to drought. Therefore, we strongly recommend that research related to the HVC should be focused for a certain period, and further investigations must be performed on the mechanisms.

Ball (2016) placed an emphasis on the models and parameters from the fitting curves and implied that the models or calculated parameters from the models need to be more practical. From the drought-rewatering experiment, we determined the lethal point, air entry point, and fastest drop point of *R*. *pseudoacacia*. We also verified that the three points can be represented by Ψ_l_, Ψ_e_, and Ψ_m_, which can be calculated from the DM, respectively. According to the Ψ values, we divided the HVC into four periods: (1) 0 ≤ Ψ < Ψ_e_, (2) Ψ_e_ ≤ Ψ < Ψ_m_, (3) Ψ_m_ ≤ Ψ < Ψ_l_, and (4) Ψ_l_ ≤ Ψ. More experimental and theoretical studies to address the HVC are urgently needed in the future to better understand the hydraulic state of the plants.

## Data Availability Statement

The original contributions presented in the study are included in the article/Supplementary Materials, further inquiries can be directed to the corresponding authors.

## Author Contributions

XL designed the research, conducted the field and laboratory measurements, and analyzed the data. ND and HW designed the research and secured funding. NW, RC, and HS contributed to the laboratory measurements and the data analysis. FW and XS conducted the data analysis. RW provided ideas for writing. XL wrote the manuscript that was intensively edited by all of the authors.

## Conflict of Interest

The authors declare that the research was conducted in the absence of any commercial or financial relationships that could be construed as a potential conflict of interest.

## Supplementary Material

Supplementary Figure 1 The visible treatments, which include: D_8_, the moderate drought group; D_12_, the severe drought group; RR, the group in which rewatering occurred until rebudding was present; and RE, the group in which rewatering occurred until the end of the experiment. The separatrix, new buds, and new leaves are marked.

## Glossary

*A*, average cross-sectional area for both ends of the stem; CDF, cumulative distribution function; CK, control group; D_3_, mild drought group; D_8_, moderate drought group; D_12_, severe drought group; D_12_L, the lower part of the sapling in the severe drought group; D_12_U, the upper part of the sapling in the severe drought group; DM, differential method; E, transpiration rate, HVC, hydraulic vulnerability curve; *K*_m_, maximum stem-specific hydraulic conductivity; *K*_s_, stem-specific hydraulic conductivity; *L*, length of the segment; *LC*, loss of conductivity; *LC*_e_, loss of conductivity at air-entry point; *LC*_l_, loss of conductivity at upper inflection point; *LC*_m_, loss of conductivity at fastest drop point; LED, light emitting diode; *m*, mass of water through the segment; *p*, intensity of the water pressure across the segment; PPFD, photosynthetic photo flux density; *Q*_m_, mass of water per unit of time through a segment; R_2_, the group of 2 days after rewatering; R_2_L, the lower part of the sapling in the group of 2 days after rewatering; R_2_U, the upper part of the sapling in the group of 2 days after rewatering; R_5_, the group of 5 days after rewatering; R_5_L, the lower part of the sapling in the group of 5 days after rewatering; R_5_U, the upper part of the sapling in the group of 5 days after rewatering; R_10_, the group of 10 days after rewatering; R_5_L, the lower part of the sapling in the group of 10 days after rewatering; R_5_U, the upper part of the sapling in the group of 10 days after rewatering; RE, the group of rewatering occurred until the end of the experiment; REL, the lower part of the sapling in the group of rewatering occurred until the end of the experiment; REU, the upper part of the sapling in the group of rewatering occurred until the end of the experiment; RR, the group of rewatering occurred until rebudding was present; RRL, the lower part of the sapling in the group of rewatering occurred until rebudding was present; RRU, the upper part of the sapling in the group of rewatering occurred until rebudding was present; *T*, time of the water conductance at maximum stem-specific hydraulic conductivity; *t*, time for the conductance measurement; TM, traditional method; *WG*, water gain; *WL*, water loss; Ψ, stem water potential; Ψ_s_, soil water potential; Ψ_e_, air-entry point; Ψ_l_, upper inflection point; Ψ_m_, fastest drop point; Ψ_12_, the pressure with a 12% hydraulic conductivity loss; Ψ_50_, the pressure with a 50% hydraulic conductivity loss; Ψ_88_, the pressure with a 88% hydraulic conductivity loss; ΔΨ, the difference between soil water potential and stem water potential.
