# Supplementary figures and images for: Quantifying Key Points of Hydraulic Vulnerability Curves From Drought-Rewatering Experiment Using Differential Method
Source: Front Plant Sci. 2021 Feb 2;12:627403. doi: 10.3389/fpls.2021.627403 (PMC7884474; doi:10.3389/fpls.2021.627403)

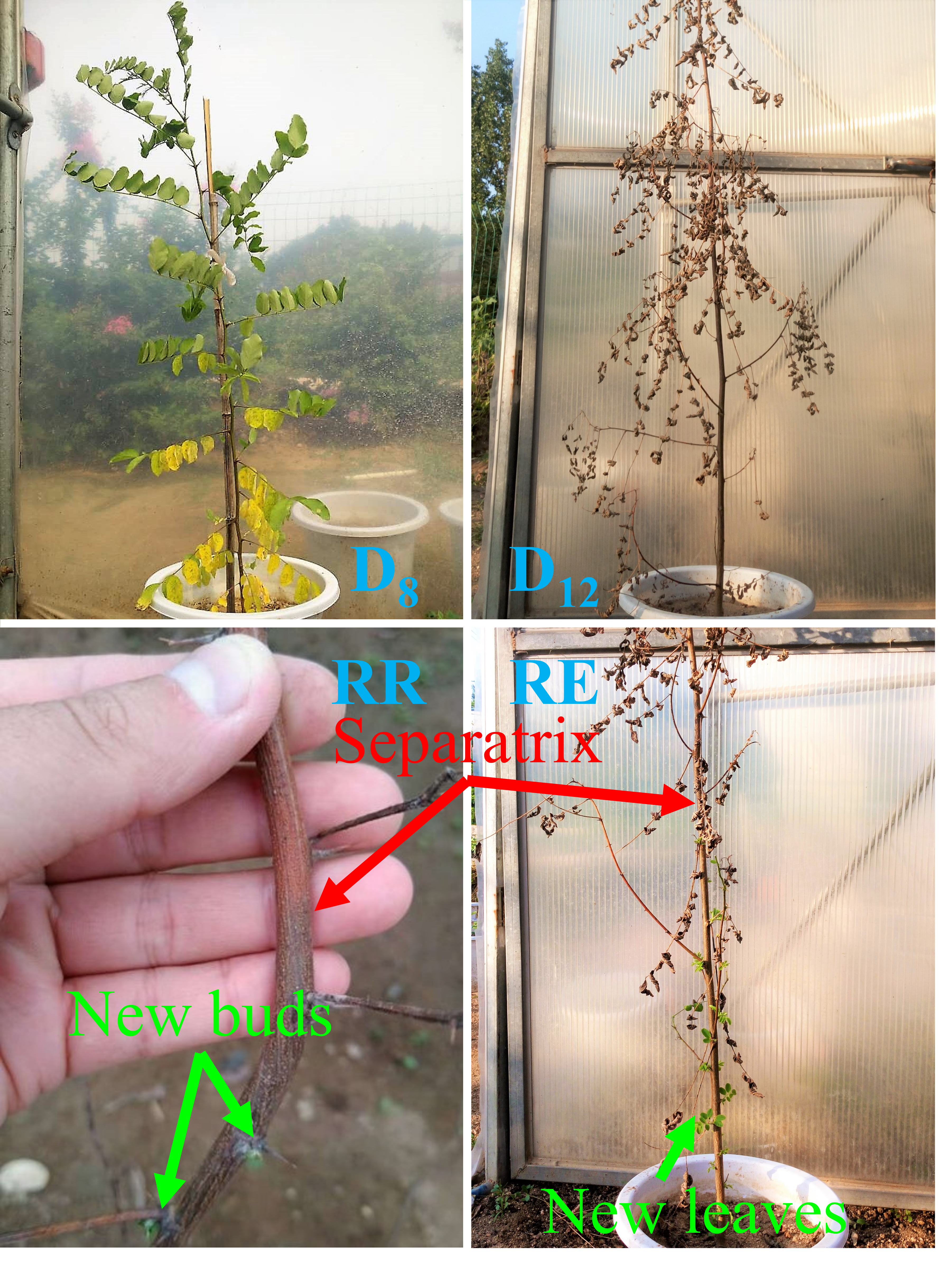

Supplement: Supplementary Figure 1 — The visible treatments, which include: D8, the moderate drought group; D12, the severe drought group; RR, the group in which rewatering occurred until rebudding was present; and RE, the group in which rewatering occurred until the end of the experiment. The separatrix, new buds, and new leaves are marked. [file Image_1.TIF]
